# Zingerone alleviates diabetic nephropathy by interrupting the ER stress-inflammation-apoptosis cascade in streptozotocin-induced diabetic mice

**DOI:** 10.1590/1414-431X2026e15216

**Published:** 2026-07-03

**Authors:** Bi Ke, Xiaojing Xiong, Chen Wang, Xiuyuan Feng, Hua Yan, Wenfeng Wang, Guang Xu

**Affiliations:** 1 Department of Cardiology, Ezhou Central Hospital, Ezhou, Hubei Province, China; 2 Department of Cardiology, Wuhan Asia General Hospital affiliated to Wuhan University of Science and Technology, Hanyang, Wuhan, Hubei Province, China

**Keywords:** Zingerone, Diabetic nephropathy, Inflammation, Endoplasmic reticulum stress

## Abstract

Endoplasmic reticulum (ER) stress plays a critical role in the pathogenesis of diabetic nephropathy. In this study, we aimed to investigate the protective effects of zingerone on the kidney and elucidate the underlying mechanisms in streptozotocin (STZ)-induced diabetic mice. Diabetic mice were randomly assigned to three groups: untreated control, diabetic control, and STZ+zingerone-treated group. Over a 3-month period, we monitored body weight, blood glucose (BG), blood urea nitrogen (BUN), serum creatinine (SCR), 24-h urinary protein (UP) excretion levels, and 24-h urinary volume (UV). Renal injury, apoptosis, inflammation, and ER stress were evaluated using histopathology, TUNEL staining, reverse transcription polymerase chain reaction (RT-PCR), western blotting, and immunofluorescence. *In vitro*, mouse glomerular mesangial cells were exposed to high glucose, with or without zingerone or the ER stress inhibitor phenylbutyric acid (PBA). Thereafter, the expression levels of ER stress markers (CHOP and GRP78), inflammatory factors (NF-κB and TGF-β1), and apoptotic indices were assessed. Diabetic mice exhibited significantly elevated BG, BUN, SCR, 24-h UP, and ER stress marker levels, along with increased 24-h UV, renal apoptosis, and inflammation. Zingerone treatment significantly mitigated these parameters and improved renal pathological manifestations. *In vitro*, both zingerone and PBA effectively suppressed high glucose-induced ER stress, inflammation, and apoptosis in mesangial cells. These findings demonstrated that zingerone attenuated diabetic renal injury through inhibition of ER stress-related pathways and downstream inflammation, thereby underscoring its potential as a therapeutic candidate for diabetic nephropathy.

## Introduction

Diabetic nephropathy (DN) represents a major microvascular complication of diabetes mellitus and a leading cause of end-stage renal disease (ESRD), posing a significant threat to patient health. Epidemiological data indicate that DN affects approximately 30-40% of individuals with diabetes (1-3). The pathogenesis of DN is multifactorial, involving glucose metabolism disorders, endoplasmic reticulum (ER) stress, oxidative stress, and hemodynamic abnormalities, all of which contribute to inflammatory tissue damage (2,3). Once progressing to ESRD, DN becomes particularly challenging to manage compared with other renal diseases due to its complex metabolic and systemic derangements. Thus, timely prevention and intervention for DN are critical to slowing disease progression.

Zingerone, a non-toxic and cost-effective natural compound derived from ginger (*Zingiber officinale*), has been reported to exhibit diverse pharmacological properties, including antioxidant, anti-inflammatory, anti-cancer, and anti-diabetic activities (4,5). Recent studies have highlighted its potential in diabetes management: zingerone has been shown to alleviate hyperglycemia and hyperlipidemia, mitigate degenerative pathological changes, and modulate oxidative stress biomarkers in diabetic models (6-11), positioning it as a promising candidate for long-term diabetes therapy.

ER stress, a homeostatic response to misfolded protein accumulation, plays a dual role in cellular physiology. While moderate ER stress serves as a protective mechanism to restore protein homeostasis, excessive or prolonged ER stress triggers maladaptive signaling cascades, including the unfolded protein response (UPR), which drives pro-inflammatory and pro-apoptotic pathways (12-15). This dysregulated ER stress has been implicated in the pathogenesis of diabetes and its complications, including DN (16,17). Accumulating evidence suggests that ER stress synergizes with inflammation and apoptosis to promote DN progression (18-24); however, the precise mechanisms underlying ER stress in DN pathophysiology remain incompletely understood, and effective ER stress-targeted therapies for DN are still lacking.

Against this backdrop, zingerone has garnered substantial research interest for its potential renoprotective effects. Preclinical studies have demonstrated that zingerone improves diabetes-induced metabolic derangements and organ damage (4-11). Nevertheless, the role of zingerone in modulating ER stress during DN development and its contribution to renal protection remain poorly characterized. Elucidating this relationship is critical for validating zingerone as a viable therapeutic option for DN. In the present study, we aimed to investigate the effects of zingerone on glycemic control, body weight, and renal function in streptozotocin (STZ)-induced diabetic mice. Additionally, we sought to clarify the underlying mechanisms by evaluating its impact on ER stress, inflammation, and apoptosis in the kidney, thereby providing foundational data to support the development of zingerone-based therapies for DN.

## Material and Methods

### Animal models

All animal experiments were approved by the Animal Care and Use Ethics Committee of Ezhou Central Hospital (approval number: 2022-0478) and were conducted in strict accordance with the NIH Guidelines for the Care and Use of Laboratory Animals. A total of 30 male C57BL/6J mice (6-8 weeks old, 20-24 g; purchased from Changzhou Cavens Experimental Animal Co., Ltd., China) were housed under specific pathogen-free conditions (25°C, 60% humidity, 12-h light/dark cycle) with *ad libitum* access to food and water. After 1 week of acclimatization, the mice were randomly divided into two initial groups: a normal control cohort (n=10) and a cohort for diabetes induction (n=20). Diabetes was induced in the model group via intraperitoneal injection of STZ (50 mg/kg per day, Sigma-Aldrich, USA) for 5 consecutive days (see Supplementary Figure S1 for the experimental timeline). Blood glucose levels were monitored weekly using tail vein blood samples (30 μL).

### Animal treatment

Following diabetes induction (fasting blood glucose ≥16.7 mmol/L), all 30 mice were allocated to the following three experimental groups for the intervention period: Control (n=10, non-diabetic), Diabetes (n=10, diabetic, vehicle-untreated), and STZ+Zingerone (n=10, diabetic, zingerone-treated, 100 mg/kg, MedChemExpress LLC, USA). Zingerone was administered via intragastric gavage once daily for 3 months. Metabolic cages were used to collect 24-h urine samples from all mice. After centrifugation of urine samples at 671 *g* for 10 min at 4°C, the supernatant was discarded, and urinary protein levels were quantified using the biuret method. For blood collection, mice were anesthetized with an intraperitoneal injection of ketamine (100 mg/kg) and xylazine (7 mg/kg) (25,26). After completion of all experiments, mice were euthanized with an overdose of sodium pentobarbital (100 mg/kg). All procedures were designed to minimize animal pain and ensure sterility. Post-blood collection, mice were provided appropriate care to maintain their welfare.

### Pathological examination of the kidney

Kidneys were fixed in 10% neutral buffered formalin for 24 h, dehydrated through a graded ethanol series (50, 70, 80, 90, 95%, and absolute ethanol), embedded in paraffin, and serially sectioned at 4-μm thickness. Sections were stained with hematoxylin and eosin (HE; MXB, China) and evaluated by two independent investigators blinded to group allocation. Histopathological parameters assessed included glomerular hypertrophy, tubular injury (epithelial flattening and cast formation), interstitial fibrosis, and inflammatory cell infiltration. Images were captured using a light microscope (Olympus IMS, USA) at ×400 magnification.

### TUNEL assay

Apoptosis was evaluated using a TUNEL staining kit (Roche, Germany) on frozen kidney sections. After DAPI counterstaining, three randomly selected cortical fields per section (×400 magnification) were imaged using a fluorescence microscope (Olympus IMS). TUNEL-positive nuclei were counted by two blinded investigators. Note that TUNEL staining may label necrotic cells, so results should be interpreted as indicators of general cell death.

### Assessment of ER stress

ER stress markers (CHOP and GRP78) were evaluated using reverse transcription polymerase chain reaction (RT-PCR), western blotting (WB), and immunofluorescence staining.

### RT-PCR

Total RNA was extracted from renal tissues/cells using the RNAsimple Total RNA Kit (Tiangen, China) following the manufacturer's protocol. Total RNA was quantified using a NanoDrop spectrophotometer (Thermo Fisher Scientific Inc., USA). RNA integrity was confirmed by agarose gel electrophoresis. For cDNA synthesis, 1 μg of total RNA was used per reaction. Complementary DNA (cDNA) was synthesized using the ReverTra Ace Real-Time Quantitative PCR (qPCR) Kit (Toyobo, Japan). RT-PCR was performed on an Applied Biosystems 7500 real-time PCR system (Thermo Fisher Scientific, Inc.). Gene expression was analyzed using the 2−ΔΔCt method and normalized to β-actin expression (27). Primers used in this study are listed in Table 1 (synthesized by GenScript Biotechnology, China).

**Table 1:** Sequences of primers used in quantitative real-time polymerase chain reaction.

Gene	Forward primer	Reverse primer
*TGF-β1*	5′-GTGTGGAGCAACATGTGGAACTCTA-3′	5′-TTGGTTCAGCCACTGCCGTA-3′
*CHOP*	5′-CCC AGG AAA CGA AGA GGA AG-3′	5′-AGT GCA GTG CAG GGT CAC AT-3′
*RP78*	5′-TTCTCAGCATCAAGCAAGGA-3′	5′-CATGGTAGAGCGGAACACGT-3′
*TNF-α*	5′-GGAGGGAGAACAGAAACTCCAG-3′	5′-CACTTGGTGGTTTGTGAGTGTG-3′
*VEGF*	5′-AATGATGAAGCCCTGGAGTG-3′	5′-TTTCTTGCGCTTTCGTTTTT-3′
*β-actin*	5′-GAGACCTTCAACACCCCAGC-3′	5′-ATGTCACGCACGATTTCCC-3′

### Western blot analysis

Protein extraction was performed using RIPA lysis buffer (Beyotime Biotechnology, China). The volume of buffer added was standardized at 150 µL per 20 mg of renal tissue and adjusted proportionally for other tissue weights to ensure consistent extraction efficiency. Following buffer addition, the samples were incubated on ice for 30 min with intermittent sonication. Sonication was performed using a probe sonicator (Shanghai Huxi Industrial Co., Ltd., China) set at 40 W (40% of maximum power) in pulsed mode (3 s on/3 s off) for 5-10 cycles. Homogenates were centrifuged at 13,800 *g* for 15 min at 4°C, and the supernatant was collected. Proteins (40 μg) were separated by sodium dodecyl sulfate-polyacrylamide gel electrophoresis (SDS-PAGE) at 80 V and transferred onto polyvinylidene difluoride (PVDF) membranes (Merck-Millipore, Germany). Membranes were blocked with 5% bovine serum albumin (BSA) for 2 h at room temperature, then probed with the primary antibodies rabbit anti-TNF-α (1:500; Santa Cruz, sc-52746; USA), rabbit anti-TGF-β1 (1:1000; Absin, abs105040; China), rabbit anti-CHOP (1:500; Santa Cruz, sc-7351), and rabbit anti-GRP78 (1:500; Santa Cruz, sc-166490) at 4°C overnight. After washing, membranes were incubated with horseradish peroxidase (HRP)-conjugated goat anti-rabbit IgG secondary antibody (1:2000; Zsbio, ZB-2301; China) at 37°C for 2 h. Gray values were quantified using Bandscan software (version 5.0; Glyko Inc.; USA). All assays included normalization to β-actin expression and were repeated three times with independent samples.

### Immunofluorescence assay

Frozen kidney tissue sections (10-μm thickness) or mesangial cells were permeabilized with 0.3% Triton X-100, blocked with 3% BSA, and incubated overnight at 4°C with the primary antibodies rabbit anti-CHOP (1:500; Santa Cruz, sc-7351) and rabbit anti-GRP78 (1:500; Santa Cruz, sc-166490). Sections/cells were then incubated with Alexa Fluor 488-conjugated goat anti-rabbit IgG secondary antibody (1:2000; Zsbio, ZF-0311) at 37°C for 2 h. Nuclei were counterstained with DAPI (1:5000; Sigma; D9542,USA). Images were acquired at ×400 magnification using an Olympus IMS microscope.

### Mouse glomerular mesangial cell culture

Primary mouse glomerular mesangial cells (MCs; catalog number CP-M057; Procell, China) were cultured in Dulbecco's modified Eagle medium (DMEM; Gibco; Thermo Fisher Scientific, Inc., USA) supplemented with 10% fetal bovine serum (FBS) at 37°C in a humidified atmosphere of 5% CO_2_. Cells were divided into five groups: normal glucose (5.5 mM), high glucose (35 mM), osmotic control (35 mM mannitol), high glucose + zingerone (1 mM), and high glucose + phenylbutyric acid (PBA; an ER stress inhibitor, 10 μM). After 48 h of incubation (when cell confluence reached ≥80%), cells were harvested for WB and RT-PCR analysis. All experiments were performed in three independent biological replicates, with three technical replicates per experiment.

### Statistical analysis

Data are reported as means±SD and derived from at least three independent experiments. Statistical comparisons between two groups were performed using the independent-samples *t*-test. For comparisons among multiple groups, one-way analysis of variance (ANOVA) followed by Tukey's *post hoc* test was used. Analyses were conducted using SPSS software (version 17.0; IBM SPSS Statistics, USA). Results with P<0.05 were considered statistically significant.

## Results

### Physiological and renal functional changes in diabetic mice

**Figure 1:**
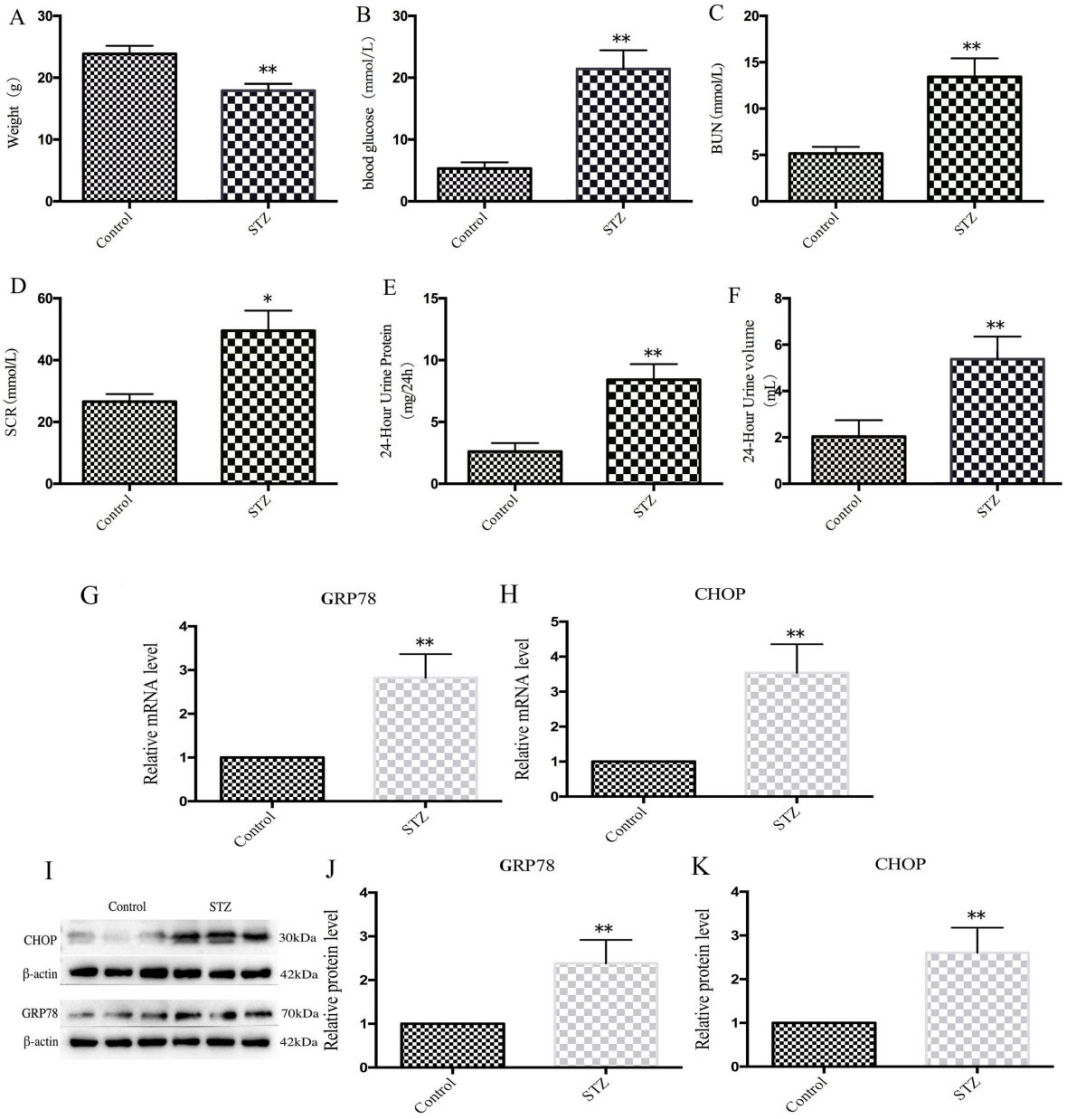
Metabolic and renal functional parameters and endoplasmic reticulum (ER) stress marker expression in normal *vs* streptozotocin (STZ)-induced diabetic mice. **A**, Body weight; **B**, blood glucose; **C**-**F**, renal dysfunction parameters, including blood urea nitrogen (BUN), serum creatinine (SCR), 24-h urine protein, and 24-h urine volume. **G** and **H**, mRNA expression levels of ER stress markers GRP78 and CHOP in diabetic mice. **I**, Representative western blot image showing ER stress-related protein expression (GRP78 and CHOP) in renal tissues from normal and diabetic mice. **J** and **K**. Quantitative analysis of GRP78 (**J**) and CHOP (**K**) protein levels, normalized to β-actin. Data are reported as means±SD (n=3). *P<0.05, **P<0.01 *vs* control group; Student's *t*-test.

STZ administration induced severe metabolic and renal abnormalities in mice. Compared with the control group, diabetic mice exhibited significantly reduced body weight (Figure 1A: 23.86±1.32 *vs* 17.90±1.12 g), marked hyperglycemia (Figure 1B: 21.40±3.03 *vs* 5.31±1.01 mmol/L), and renal dysfunction. Key renal parameters were significantly impaired in diabetic mice compared with controls: BUN level (Figure 1C: 13.42±2.00 *vs* 5.17±0.71 mg/dL), SCR level (Figure 1D: 49.52±6.52 *vs* 26.60±2.45 μmol/L), 24-h UP level (Figure 1E: 8.42±1.27 *vs* 2.61±0.69 mg/24 h), and 24-h UV (Figure 1F: 5.38±0.97 *vs* 2.04±0.71 mL). Molecular analyses revealed upregulation of ER stress markers: mRNA (Figure 1G and H) and protein (Figure 1I-K) levels of CHOP and GRP78 were significantly higher in diabetic mice than in controls.

### Zingerone ameliorated renal pathology and apoptosis

**Figure 2:**
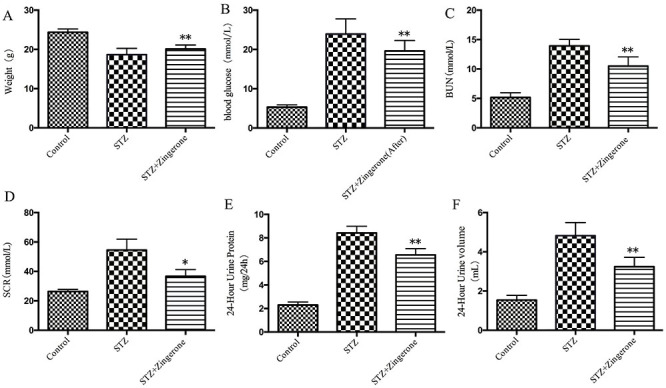
Effects of zingerone on metabolic and renal parameters in streptozotocin (STZ)-induced diabetic mice. **A**-**F**, Body weight, blood glucose level, blood urea nitrogen (BUN) level, serum creatinine (SCR) level, 24-h urine protein, and 24-h urine volume were compared among control, diabetic, and STZ+zingerone-treated groups. Data are reported as means±SD (n=5). *P<0.05,**P<0.01 *vs* STZ group; ANOVA.

**Figure 3:**
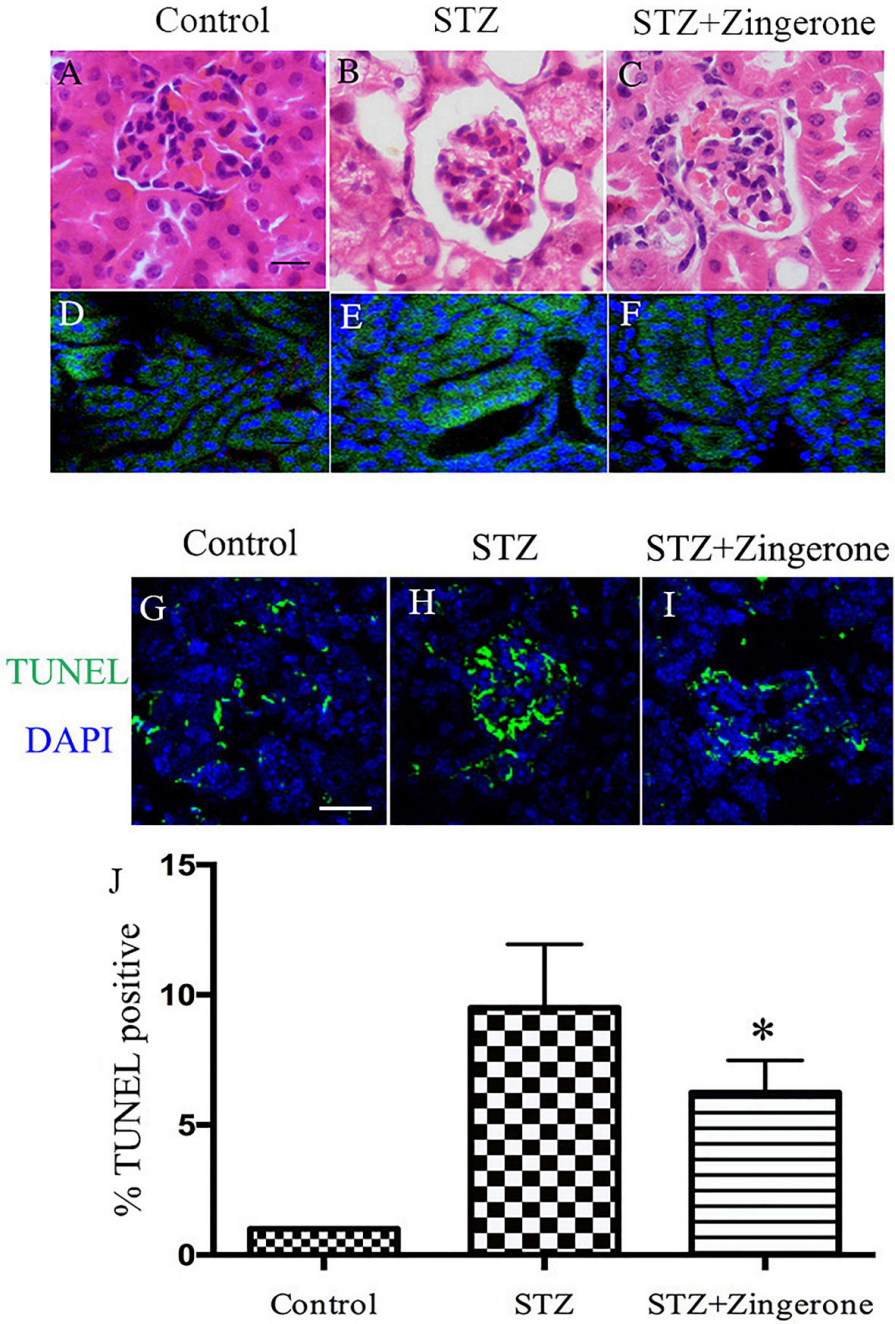
Histopathological, immunofluorescence, and cell apoptosis analyses in diabetic mice with zingerone treatment. **A**-**C**, Hematoxylin-eosin (HE) staining of renal tissues from control (**A**), diabetic (**B**), and streptozotocin (STZ)+zingerone-treated (**C**) mice. Diabetic kidneys showed glomerular mesangial proliferation, tubular epithelial vacuolation, and interstitial inflammation/fibrosis, which were alleviated by zingerone. **D**-**F**, Immunofluorescence staining of CHOP (green) in renal tissues, with DAPI (blue) for nuclear counterstaining. CHOP fluorescence intensity was reduced in zingerone-treated kidneys. **G**-**I**, Representative TUNEL-stained images of renal tissues from control, diabetic, and STZ+zingerone-treated groups. Green fluorescence indicates TUNEL-positive apoptotic cells. **J**, Quantitative analysis of apoptotic cell proportion. Zingerone treatment significantly reduced the percentage of TUNEL-positive cells compared with the diabetic group. Data are reported as means±SD (n=3). *P≤0.05 *vs* STZ group; ANOVA. Scale bar: 50 μm.

Compared with the diabetic group, zingerone treatment significantly improved body weight (Figure 2A: 20.55±0.71 *vs* 19.00±1.02 g), blood glucose level (Figure 2B: 19.60±2.69 *vs* 24.90±2.87 mmol/L), and renal function, including BUN level (Figure 2C: 10.49±1.58 *vs* 13.92±1.12 mg/dL), SCR level (Figure 2D: 36.73±5.17 *vs* 54.79±6, 30 μmol/L), 24-h UP level (Figure 2E: 6.56±0.53 *vs* 8.42±0.56 mg/24 h), and 24-h UV (Figure 2F: 3.25±0.47 *vs* 4.82±0.67 mL). In HE-stained renal sections, the control group (Figure 3A) displayed normal glomerular mesangial matrix, intact basement membrane, well-arranged renal tubular epithelial cells with uniform cytoplasm and nuclei, and no interstitial inflammation or fibrosis. The STZ group (Figure 3B) exhibited glomerular mesangial proliferation, basement membrane thickening, tubular epithelial vacuolation/edema, and interstitial inflammatory infiltration/fibrosis. Notably, the STZ+zingerone group (Figure 3C) showed significantly ameliorated glomerular and tubular lesions, with reduced interstitial changes, consistent with zingerone's protective effect. CHOP fluorescence intensity (Figure 3D-F) was also consistent with these HE findings. TUNEL assay results for cell apoptosis revealed a 34% reduction in the apoptotic cell proportion (Figure 3G-J; STZ: 9.50±2.45% *vs* STZ+zingerone: 6.24±1.24%).

### Modulation of ER stress and inflammatory pathways

**Figure 4:**
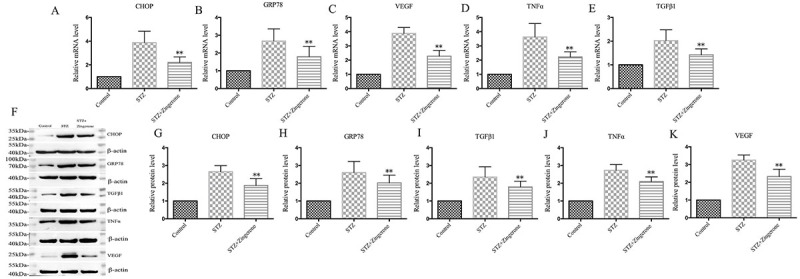
Zingerone modulated inflammatory and endoplasmic reticulum (ER) stress pathways in diabetic kidneys. **A**-**E**, RT-PCR analysis of mRNA expression levels of ER stress markers (CHOP and GRP78; **A** and **B**), VEGF (**C**), and inflammatory cytokines (TNF-α and TGF-β1; **D** and **E**) in kidneys of control, diabetic (STZ), and streptozotocin (STZ)+zingerone-treated animals. **F**, Representative western blot image showing protein expression of CHOP, GRP78, TGF-β1, TNF-α, and VEGF. **G**-**K**, Quantitative analysis of protein levels normalized to β-actin. Zingerone treatment downregulated both inflammatory and ER stress markers. Data are reported as means±SD (n=3). **P<0.01 *vs* STZ group; ANOVA.

*In vivo*, zingerone treatment suppressed the expression of ER stress markers in the kidneys of diabetic mice. Levels of CHOP and GRP78 mRNA (Figure 4A and B) and protein (Figure 4F-H) in STZ+zingerone-treated mice were significantly lower than those in the diabetic group. Additionally, levels of inflammatory cytokines (TNF-α and TGF-β1) and VEGF were reduced in STZ+zingerone-treated mice (Figure 4C-E and 4I-K).

**Figure 5:**
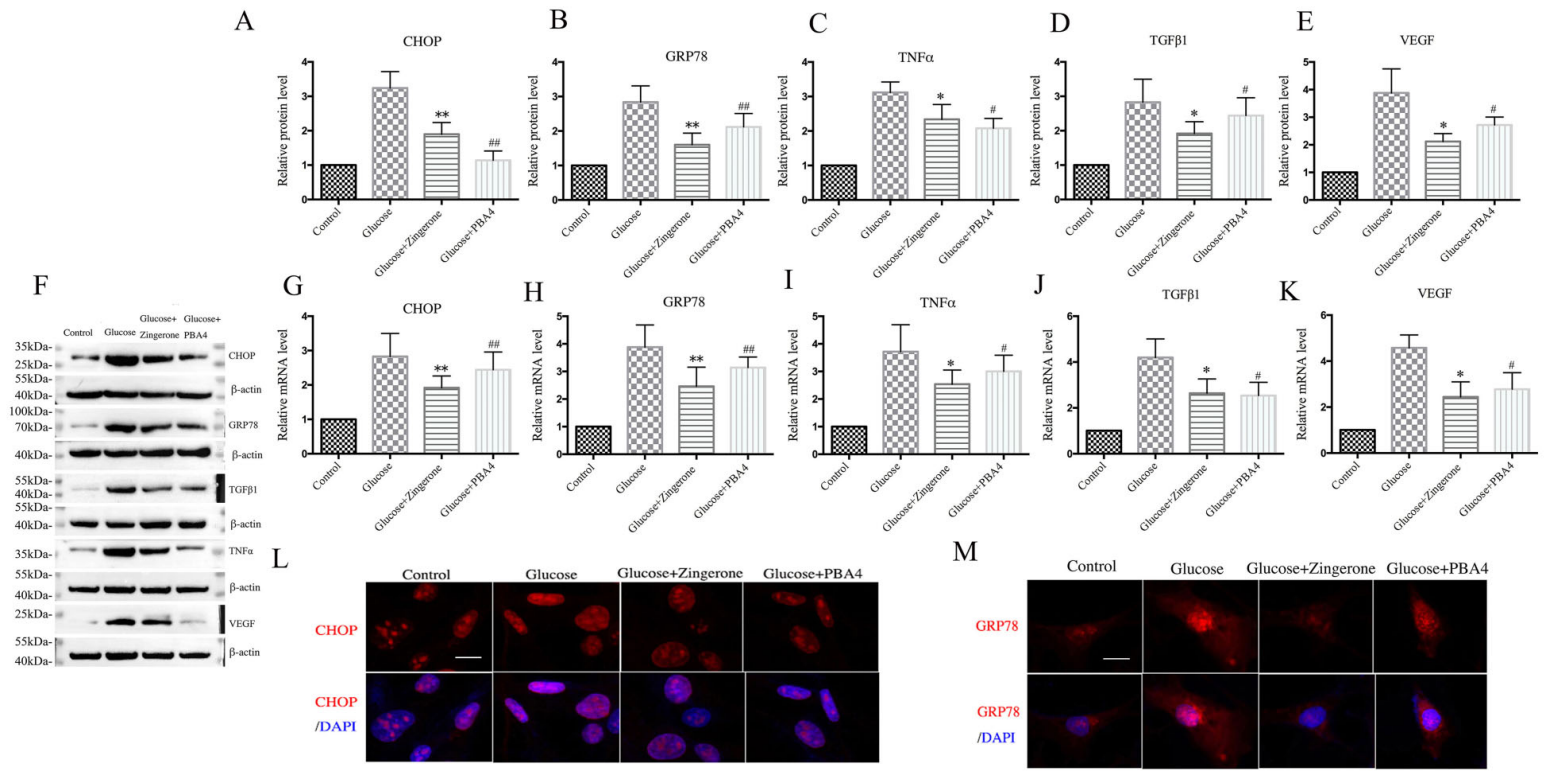
Zingerone and phenylbutyric acid (PBA) suppressed high-glucose-induced endoplasmic reticulum (ER) stress in mouse glomerular mesangial cells. **A**-**E**, mRNA expression levels of ER stress markers (CHOP, GRP78; **A** and **B**), inflammatory cytokines (TNF-α, TGF-β1; **C** and **D**), and VEGF (**E**) in high-glucose-exposed mesangial cells treated with zingerone or PBA. **F**, Representative western blot image of CHOP, GRP78, TNF-α, TGF-β1, and VEGF. **G**-**K**. Quantitative analysis of protein levels normalized to β-actin. **L** and **M**, Immunofluorescence staining of CHOP (red) and GRP78 (red) in mesangial cells (scale bar: 50 μm). Data are reported as means±SD (n=3). *P<0.05, **P<0.01, ^#^P<0.05, ^##^P<0.01 *vs* high glucose group; ANOVA.

*In vitro*, in high-glucose-exposed mouse glomerular mesangial cells, both zingerone and PBA significantly reduced CHOP and GRP78 mRNA (Figure 5A and B) and protein expression (Figure 5F-H). Expression of inflammatory cytokines (TNF-α and TGF-β1) and VEGF was also downregulated following zingerone and PBA treatment (Figure 5C-F and 5I-K; P<0.05). Furthermore, zingerone and PBA treatment reduced CHOP and GRP78 fluorescence intensity (Figure 5L and M).

## Discussion

DN remains a severe complication of diabetes mellitus, with ER stress emerging as a critical pathological driver (1-3, 16-23). Clinically, elevated levels of GRP78 and oxygen-regulated protein 150 (ORP150) in the renal tissues of DN patients have been observed, indicating that ER stress activation correlates with the severity of renal injury (23). Our findings demonstrated that zingerone, a bioactive compound derived from ginger, exerted significant renal protective effects in STZ-induced diabetic mice by suppressing the PERK-eIF2α-CHOP axis of ER stress pathway and its downstream inflammatory cascade. This novel mechanistic insight highlights zingerone's action at the convergence of proteotoxic stress and apoptotic cell death.

Notably, CHOP, a key transcription factor in ER stress-mediated apoptosis, directly binds to the TRB3 gene promoter, inhibits Akt activation, and induces apoptosis (18). The consistent reduction in CHOP expression observed in both mouse renal tissues and high-glucose-exposed mesangial cells underscores zingerone's ability to disrupt the PERK-mediated phosphorylation cascade that triggers this terminal effector of ER stress-induced apoptosis. Mechanistically, CHOP serves as a molecular linchpin linking ER dysfunction to mitochondrial apoptosis via Bim activation and caspase-3 cleavage, which aligns with the 37% reduction in TUNEL-positive cells in zingerone-treated mice. GRP78, a major early signaling factor of the UPR, binds to ER stress receptors and marks the initiation of ER stress (14,26). The complementary suppression of GRP78 expression suggests that zingerone may mitigate ER stress, potentially through modulation of the chaperone system or reduction of oxidative stress, though direct interaction with ER stress sensors remains to be experimentally verified.

The translational consistency between our whole-animal and cellular models strengthens the biological validity of these findings. In diabetic mice, zingerone not only improved hallmark renal dysfunction parameters, including a 25% reduction in BUN, 33% reduction in SCR, and 22% reduction in proteinuria, but also reversed histopathological hallmarks such as glomerular hypertrophy and tubular damage. These *in vivo* effects were mirrored in glomerular mesangial cells, where zingerone exhibited efficacy comparable to the established ER stress inhibitor PBA in blocking high-glucose-induced CHOP/GRP78 overexpression. Given the pivotal role of mesangial cells in diabetic glomerulosclerosis (where their ER stress response drives matrix expansion and cytokine production), this cross-model concordance is physiologically significant, confirming that mesangial pathophysiology effectively recapitulates core features of DN and validates our experimental approach.

Inflammation is another critical driver of DN progression, with excessive inflammatory responses causing severe renal cell damage. For example, NF-κB, a key TNF-α-mediated transcription factor, regulates inflammatory pathways, immune responses, cell proliferation, and apoptosis, and plays an imperative role in DN pathogenesis (27-34). NF-κB activation has been observed in both diabetic patients and animal models (30), and studies have shown that inhibiting NF-κB reduces renal vascular damage and podocyte death in diabetes, underscoring its essential role in DN development (30,31).

In the context of existing literature, our study advances key insights. While Rehman et al. (34) and Cui et al. (35) reported the antioxidant and anti-inflammatory effects of zingerone in diabetic kidneys, they did not explore ER stress pathways. Similarly, Akaras et al. (36) focused on arsenic-induced nephrotoxicity rather than diabetes-specific mechanisms. This work is the first to: 1) identify the PERK-CHOP signaling axis as the primary molecular target of zingerone, moving beyond its known antioxidant effects; 2) demonstrate synchronized efficacy in both *in vivo* and *in vitro* models using quantitative endpoints; and 3) demonstrate that the amelioration of renal function is tightly associated with the suppression of ER stress. These findings collectively highlight zingerone's action as a targeted modulator of the ER stress pathway.

Methodologically, while our approach successfully delineated the association of zingerone with the PERK-CHOP axis, several considerations merit attention. The absence of direct PERK phosphorylation measurements limited mechanistic granularity; however, the coordinated suppression of the downstream effector eIF2α (via CHOP) strongly implied PERK involvement. Our 12-week intervention period captured early-to-mid stage DN but not end-stage fibrosis. Additionally, the role of tubular ER stress, given its contribution to tubular atrophy and disease progression, warrants further investigation. Future studies using PERK-knockout models could confirm target specificity, and exploring synergies with SGLT2 inhibitors might uncover combinatorial therapeutic potential.

**Figure 6:**
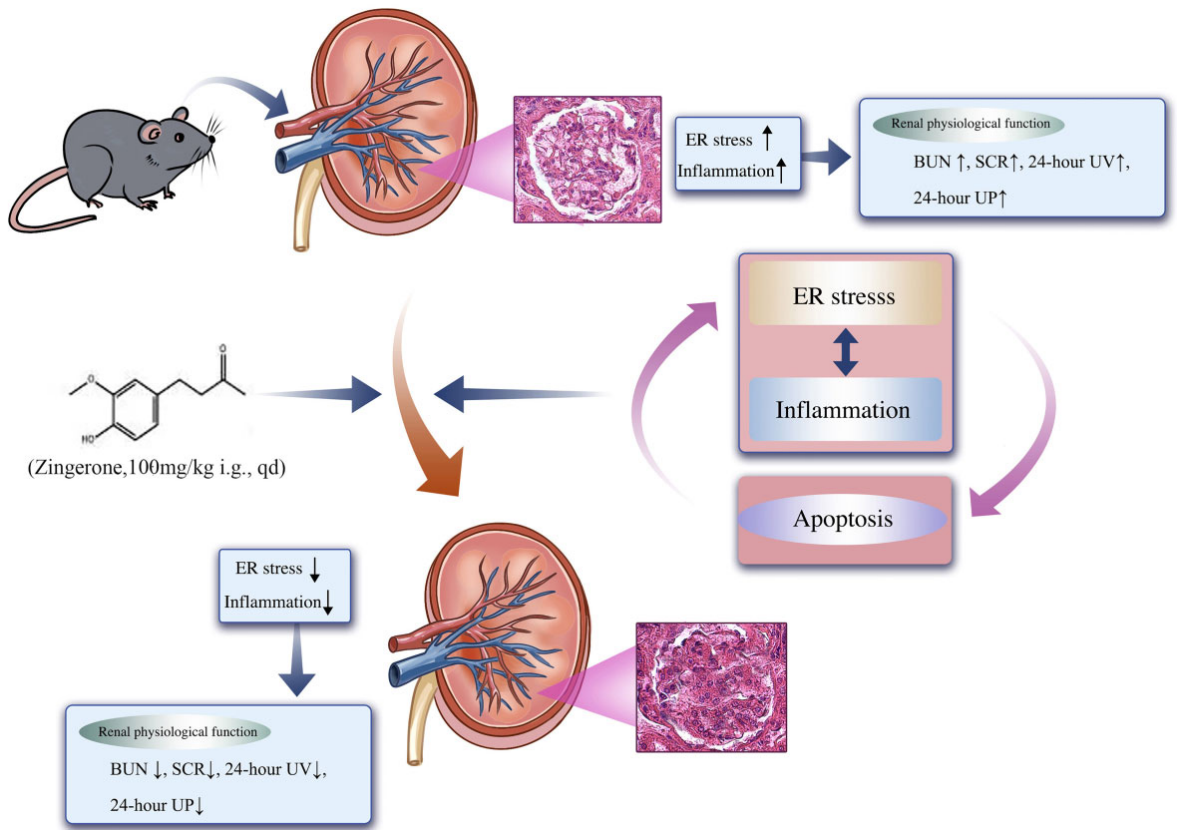
Mechanistic summary: zingerone attenuated diabetic nephropathy via endoplasmic reticulum (ER) stress inhibition. Zingerone protected against diabetic kidney injury by suppressing the PERK-eIF2α-CHOP ER stress axis, which in turn reduced downstream inflammation (NF-κB, TNF-α, TGF-β1) and apoptosis (TUNEL-positive cells). This dual effect improved renal metabolic function [blood urea nitrogen (BUN), serum creatinine (SCR), 24-h urinary volume (UV), and urinary protein (UP)] and preserved renal architecture.

In conclusion, this research elucidated a previously unrecognized mechanism by which zingerone protected against DN: selective inhibition of the PERK-eIF2α-CHOP ER stress pathway. By disrupting this signaling cascade, zingerone concurrently attenuated inflammation and apoptotic cell death, ultimately preserving renal architecture and function (Figure 6). The striking concordance of these effects across whole-animal physiology and cellular models underscores their pathophysiological relevance, with mesangial cells emerging as critical therapeutic targets. Given zingerone's natural origin, low toxicity, and multi-target efficacy, these findings advocate for its development as an adjunct therapy for DN, particularly in early intervention strategies aimed at halting ER stress-mediated damage before irreversible fibrosis ensues.

## Data Availability

All data generated or analyzed during this study are included in this published article.

## Supplementary Materials

Supplementary Material Click to view [pdf].
