# Supplementary figures and images for: Zingerone alleviates diabetic nephropathy by interrupting the ER stress-inflammation-apoptosis cascade in streptozotocin-induced diabetic mice
Source: Braz J Med Biol Res. 2026 Jul 3;59:e15216. doi: 10.1590/1414-431X2026e15216 (PMC13331251; doi:10.1590/1414-431X2026e15216)

**Figure S1.** Experimental timeline. STZ: streptozotocin.

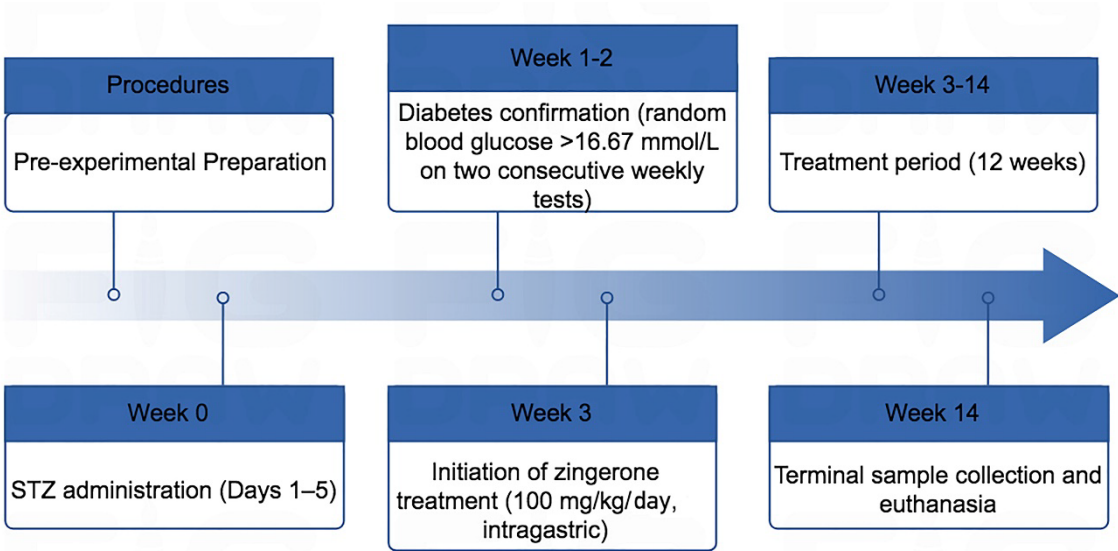

Supplement: Supplementary Material [file 1414-431X-bjmbr-59-e15216-suppl.pdf]
